# Effectiveness of a novel ozone and hydrogen peroxide gas-vapour system for the rapid high level disinfection of surfaces and healthcare spaces

**DOI:** 10.1186/1753-6561-5-S6-O38

**Published:** 2011-06-29

**Authors:** D Zoutman, M Shannon, K Brown

**Affiliations:** 1 Medical Microbiology, Queen’s University, Ontario, Canada; 2 Medizone International Inc, Kingston, Ontario, Canada

## Introduction / objectives

Vapour based fumigant systems for disinfection of healthcare surfaces and spaces are an evolving technology. A new system that uses an ozone based process to create a highly reactive oxidative gas-vapour mixture that is noncorrosive was tested in vitro and in vivo for antimicrobial disinfection effectiveness.

## Methods

Ozone gas at 80 parts per million (ppm) was combined with 1% stabilized hydrogen peroxide vapour at 80% relative humidity in a test chamber and upscaled to a 82 cubic meter room using 3.75% hydrogen peroxide at 30 minutes. Test organisms included methicillin resistant *S. aureus*, vancomycin resistant Enterococcus, *E. coli*, *P. aeruginosa*, and *C. difficile* spores dried onto stainless steel discs.

## Results

The combination of 80 ppm ozone with 1% hydrogen peroxide vapour in the test chamber achieved a very high level of disinfection of at least 6 log_10_ reduction of the bacteria and *C. difficile* spores tested on steel discs during a 15 minute exposure. The entire system was scalable such that it achieved the same high level of disinfection of an 81 cubic meter room in 30 minutes with 3.75% hydrogen peroxide and 80 PPM of ozone against MRSA and *C. difficile* spores.

## Conclusion

The ozone and hydrogen peroxide gas-vapour mixture provides a very rapid and high level of disinfection of steel surfaces against important healthcare associated bacterial pathogens. The system is an advanced oxidative process providing a rapid and effective means to disinfect healthcare surfaces and spaces to a very high level, particularly against *C. difficile* spores.

## Disclosure of interest

D. Zoutman Grant/Research support from Medizone International Inc, Shareholder of Medizone International Inc, M. Shannon Employee of Medizone International Inc, Shareholder of Medizone International Inc, K. Brown Employee of Medizone International Inc.

